# Human adenosine deaminase type 2 deficiency enhances NK cell activation but impairs maturation and function

**DOI:** 10.1172/JCI196381

**Published:** 2025-11-04

**Authors:** Jarne Beliën, Amber De Visscher, Bethany Pillay, Marjon Wouters, Verena Kienapfel, Eline Bernaerts, Tania Mitera, Nele Berghmans, Bénédicte Dubois, Leen Moens, Patrick Matthys, Isabelle Meyts

**Affiliations:** 1 Laboratory for Neuroimmunology, Department of Neurosciences, Leuven Brain Institute,; 2 Laboratory of Immunobiology, Department of Microbiology, Immunology and Transplantation, Rega Institute for Medical Research,; 3 Laboratory of Inborn Errors of Immunity, Department of Microbiology, Immunology and Transplantation, and; 4 Laboratory of Molecular Immunology, Department of Microbiology, Immunology and Transplantation, Rega Institute for Medical Research, KU Leuven, Leuven, Belgium.; 5 Department of Neurology and; 6 Department of Pediatrics, University Hospitals Leuven, Leuven, Belgium.

**Keywords:** Immunology, Inflammation, Innate immunity, Monogenic diseases, NK cells

## Abstract

NK cells, or natural killer cells, might be interesting players to investigate further in the disease process of patients with deficiency of ADA2, a rare, recently discovered inborn error of immunity.

**To the Editor:** Human adenosine deaminase type 2 (ADA2) deficiency (DADA2) is an inborn autoinflammatory disease (global prevalence of 1 in 222,000) with heterogeneous clinical manifestations, including vasculitis, early-onset stroke, cytopenia, bone marrow failure, and immunodeficiency. It results from deleterious biallelic mutations in the *ADA2* gene, which encodes a dimeric adenosine deaminase that is highly expressed across the immune system, with strongest surface binding on neutrophils, monocytes, B cells, and NK cells (1). The effect on immune cells remains incompletely understood. Initial reports showed a shift toward proinflammatory myeloid subsets (2) and perturbations in the lymphoid compartment including blocked B cell development, a skewed B cell repertoire, impaired T cell memory, T cell exhaustion, reduced NK cell numbers, and a shift toward immature CD56^bright^ NK cells (2–4).

Here, we immunophenotyped NK cells in peripheral blood of 11 patients with DADA2 (Supplemental Table 1; supplemental material available online with this article; https://doi.org/10.1172/JCI196381DS1). First, we confirmed diminished NK cell frequencies in DADA2 versus healthy controls (HCs) (Figure 1, A and C), with a trend toward an increased CD56^bright^/CD56^dim^ ratio in DADA2 (Figure 1, B and C) and a decrease in the expression of the terminal NK cell maturation marker CD57 (Figure 1C), indicating a less mature NK cell compartment in DADA2.

Next, we found canonical activation markers to be upregulated among NK cells in DADA2 (Figure 1D). Notably, activation was most pronounced in patient 2, who later underwent hematopoietic stem cell transplantation (HSCT) for refractory disease. NK cell activation is regulated by activating and inhibitory receptors (5). Of all investigated receptors, only inhibitory killer Ig–like receptors (KIRs) were significantly downregulated in DADA2 (Supplemental Figure 2A). NK-activating ligands ULBPs were increased on T cells in DADA2 (Figure 1E), which may explain the enhanced NK cell activation. In line with the phenomenon of lymphocyte exhaustion after persistent activation, there was a clear trend toward an exhausted NK cell phenotype in DADA2 (Supplemental Figure 2B).

Given the loss of terminally differentiated, cytotoxic CD56^dim^ NK cells in DADA2, we investigated the expression of NK cell cytotoxic mediators (5). Perforin and granzyme A, but not granzyme B, were decreased in CD56^dim^ NK cells but increased in CD56^bright^ NK cells, which are classically regarded as less cytotoxic and more regulatory in nature (Figure 1F and Supplemental Figure 2C) (5). DADA2 NK cells exhibited increased ex vivo degranulation as evidenced by surface expression of CD107a (Figure 1F and Supplemental Figure 2C), which was confirmed in vitro by measuring degranulation of isolated NK cells after a 20-hour culture with or without K562 target cells (Figure 1G). Interestingly, a patient who was resampled after HSCT with 90% donor chimerism showed degranulation within the normal range, while 70% donor chimerism in another patient led to a degranulation level within the range of the nontransplanted patients. Finally, DADA2 NK cells also exhibited significantly reduced cytotoxicity against K562 cells (Figure 1H), likely due to the loss of important cytotoxic mediators such as perforin and granzyme A. Notably, 1 patient (patient 11) retained near-normal NK cell cytotoxicity, highlighting heterogeneity among the patients. NK cells alternatively induce target cell death through death ligands such as TNF-related, apoptosis-inducing ligand (TRAIL), a mechanism mainly used by CD56^bright^ NK cells (5). Strikingly, TRAIL was significantly increased on both CD56^bright^ and CD56^dim^ DADA2 NK cells (Figure 1I and Supplemental Figure 2D), suggesting a compensatory increase in TRAIL cytotoxic pathway activity. Whether this provides a potential mechanism for the increased spontaneous immune cell death observed in DADA2 (6) constitutes an interesting avenue for future research.

Last, we observed a trend toward increased TNF production by NK cells in DADA2, even in unstimulated conditions, although statistical significance was not reached (Supplemental Figure 2E). This observation warrants further investigation, given the characteristic perivascular deposition of TNF in patients with DADA2, the role of TNF in immune cell death, and the clinical use of TNF inhibitors (1).

Here, we report an altered phenotype and functional impairment of NK cells in DADA2. Given the occurrence of NK cell–related clinical features in DADA2, such as recurrent viral infections, macrophage activation syndrome, and malignancies (1, 2), and given the interindividual heterogeneity in which some patients with DADA2 have near-normal NK cell phenotypes, our study provides a rationale for further in-depth investigation of the contribution of NK cells to the heterogeneous disease spectrum in DADA2.

## Funding support

- Research Foundation-Flanders (FWO-Vlaanderen, 11A0523N/11A0525N, PhD Fellowship, to JB).
- Research Foundation-Flanders (FWO-Vlaanderen, grant G0B5120N, to IM).
- Jeffrey Modell Foundation (to IM).
- European Research Council (ERC) under the European Union’s Horizon 2020 research and innovation programme (grant agreement No. 948959).
- European Reference Network for Rare Immunodeficiency, Autoinflammatory and Autoimmune Diseases.
- FWO-Vlaanderen grant 1805826N (to IM).

## Supplementary Material

Supplemental data

Supporting data values

## Figures and Tables

**Figure 1:**
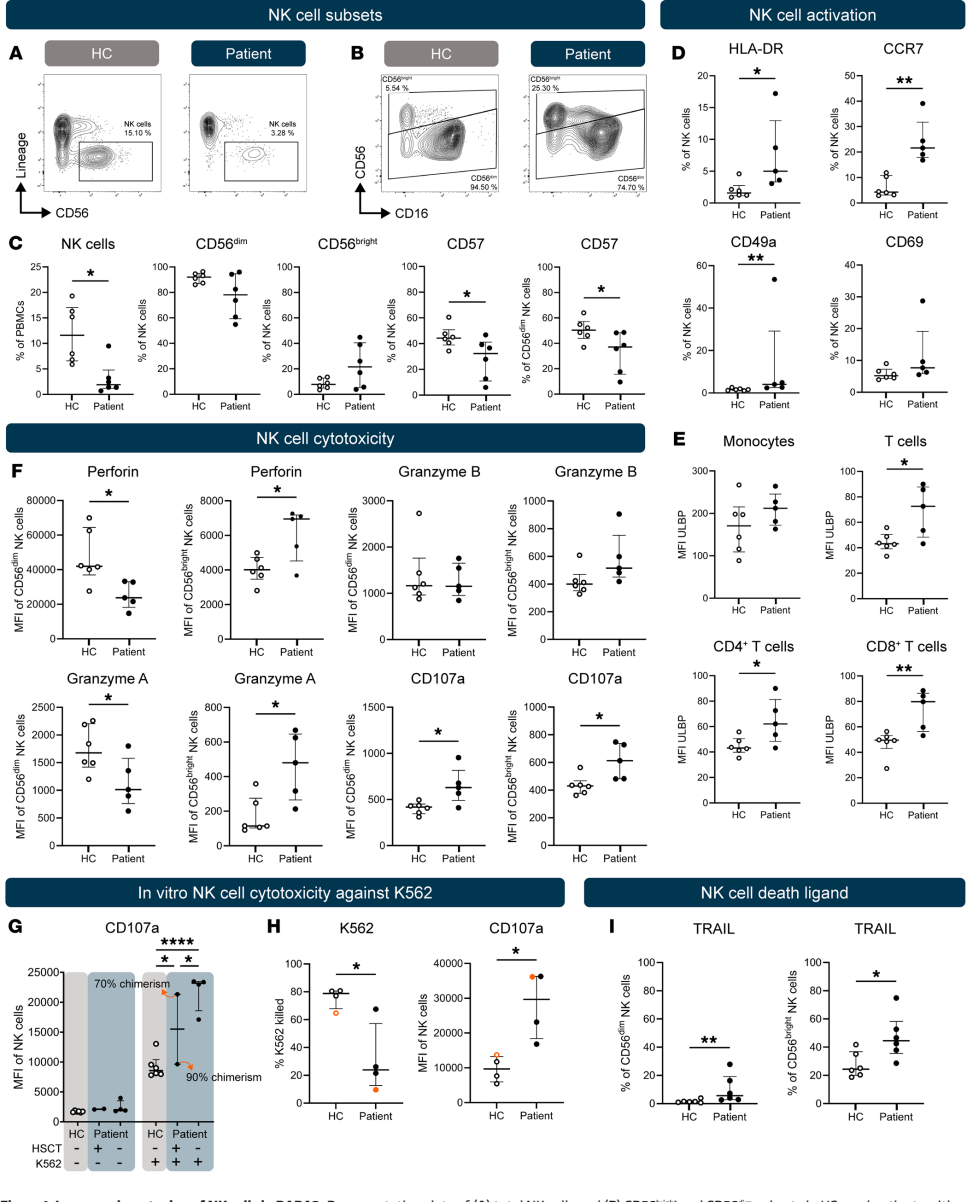
Immunophenotyping of NK cells in DADA2. Representative plots of (**A**) total NK cells and (**B**) CD56^bright^ and CD56^dim^ subsets in HCs and patients with DADA2. (**C**–**F** and **I**) Ex vivo percentage or median fluorescence intensity (MFI). (**G**) MFI of CD107a on isolated NK cells after 20 hours of culturing with or without K562 cells. (**H**) Percentage of dead K562 cells, corrected for background apoptosis and CD107a MFI on NK cells after 4 hours of coculturing. Orange dots refer to patient 8 and HC 8, where the assay used a lower effector/target ratio due to sample limitations (2.4:1 vs. 4:1). Each dot represents an individual; bars show the median with the IQR. *P < 0.05, **P < 0.01, and ****P < 0.0001, by 2-tailed, unpaired *t* test or Mann-Whitney *U* test (the exact test used is indicated in Supplemental Methods) (**C**–**F**, **H**, and **I**), or 1-way ANOVA with Šídák’s test (**G**). The complete gating strategy is shown in Supplemental Figure 1.

## Version 1 11/04/2025

In-Press Preview

## Version 2 01/02/2026

Electronic publication
