# Supplementary material for: Human adenosine deaminase type 2 deficiency enhances NK cell activation but impairs maturation and function
Source: J Clin Invest. 2025 Nov 4;136(1):e196381. doi: 10.1172/JCI196381 (PMC12721890; doi:10.1172/JCI196381)
Supplement: Supplemental data [file jci-136-196381-s275.pdf]

HUMAN ADENOSINE DEAMINASE TYPE 2 DEFICIENCY: ENHANCED NK CELL ACTIVATION BUT IMPAIRED MATURATION AND FUNCTION

SUPPLEMENTARY TABLES & FIGURES

Supplementary Table 1. Characteristics of DADA2 cohort

	Sex	Age at sampling (years)	ADA2 genotype	Predominant clinical phenotype	Treatment at sampling
Patient 1	Male	11	c.973-2A>G / c. del1240-1442	Vasculitis, hypogammaglobulinemia, hepatosplenomegaly	SCIG, ibuprofen
Patient 2	Male	28	c.140G>T / c. del1240-1442	Bone marrow failure, hypogammaglobulinemia, hepatosplenomegaly	SCIG, etanercept, filgrastim / *resampled post-HSCT: SCIG tapering*
Patient 3	Male	27	c.140G>T / c.140G>T	Hypogammaglobulinemia, hepatosplenomegaly	IVIG, etanercept
Patient 4	Female	5	c.140G>T / c.506G>A	Hypogammaglobulinemia, hepatosplenomegaly, stroke	Etanercept
Patient 5	Female	9	c.140G>T / c.506G>A	Bone marrow failure, hypogammaglobulinemia, hepatosplenomegaly	SCIG, etanercept, filgrastim (daily), Neoral / *resampled post-HSCT: filgrastim (weekly), amoxicillin*
Patient 6	Female	9	c.139G>A / c.139G>A	Vasculitis	Etanercept
Patient 7	Female	17	c.973-2T>C / c.del1240-1442	Vasculitis, hypogammaglobulinemia, arthralgia, warts	Etanercept
Patient 8	Female	53	c.973-2ª>G/c.506G>A(p.Arg169Gln)	Vasculitis, neutropenia, hypogammaglobulinemia	Filgrastim
Patient 9	Male	29	c.140G>T p.Gly47Val/c.140G>T p.Gly47Val	Thrombocytopenia, hypogammaglobulinemia	SCIG, etanercept

Patient 10	Female	9	c.140G>T (p.Gly47Val)/ c.506G>A(p. Arg169Gln)	Vasculitis, neutropenia, hypogammaglobulinemia, stroke	SCIG, etanercept, hydroxychloroquine, ruxolitinib
Patient 11 (Brother of Patient 7)	Male	15	c.973-2T>C / c. del1240-1442	Vasculitis, neutropenia, hypogammaglobulinemia	Etanercept

SCIG, subcutaneous immunoglobulin; HSCT, hematopoietic stem cell transplant; IVIG, intravenous immunoglobulin

Supplementary Table 2. Human monoclonal fluorochrome-labeled antibodies

Antigen	Fluorochrome	Clone	Company
Immunophenotyping			
CD3	Percp-Cy5.5	SK7	Life Technologies
CD3	APC-eF780	SK7	Life Technologies
CD4	BV785	OKT4	BioLegend
CD8A	APC-R700	RPA-T8	BD Biosciences
CD14	Percp-Cy5.5	61D3	Life Technologies
CD16	BUV395	3G8	BD Biosciences
CD19	Percp-Cy5.5	HIB19	BioLegend
CD49a	PE Cy7	TS2/7	BioLegend
CD56	BV421	NCAM16.2	BD Biosciences
CD57	PE Cy7	TBO1	Life Technologies
CD69	APC-R700	FN50	BD Biosciences
CD94/KLRD1/KP43	PE Cy7	DX22	BioLegend
CD95/Fas	BV711	DX2	BD Biosciences
CD107a/LAMP1	PE	H4A3	BioLegend
CD123	Percp-Cy5.5	6H6	BioLegend
CD197/CCR7	BV711	G043H7	BioLegend
CD226/DNAM-1	BV786	11A8	BioLegend
CD279/PD1	BB515	EH12.1	BD Biosciences
Granzyme A	PE	CB9	Life Technologies
Granzyme B	BV510	GB11	BD Biosciences
Granzyme K	EF660	G3H69	Life Technologies
HLADR	BV510	G46-6	BD Biosciences
IFN-y	APC	4S.B3	Life Technologies
KIR/CD158a	PE	HP-3E4	BD Biosciences
KIR/CD158b1/b2/j	PE	CH-L	BD Biosciences
KIR/CD158e1/e2	PE	Z27.3.7	Beckman Coulter
KIR/CD158g	AF647	1165A	R&D systems
KIR/CD158h	AF647	1127B	R&D systems
KIR/CD158i	AF647	179315	BD Biosciences
NKG2A/CD159a	PE Cy7	S19004C	BioLegend
NKG2D/CD314	BV510	1D11	BD Biosciences
NKp30/CD337	BV711	P30-15	BD Biosciences

NKp44/CD336	BV786	p44-8	BD Biosciences
NKp46/CD335	BV510	9E2/NKp46	BD Biosciences
Perforin	AF488	δG9	BD Biosciences
TIGIT	BV786	741182	BD Biosciences
TNFA	PE	MAb11	Life Technologies
TRAIL/CD253	PE	TRAIL	Life Technologies
ULBP-1	PE	170818	R&D systems
ULBP-3	PE	166510	R&D systems
ULBP-4	PE	709116	R&D systems
ULBP-2/5/6	PE	165903	R&D systems
NK cell cytotoxicity assay			
CD3	Percp-Cy5.5	SK7	Life Technologies
CD14	Percp-Cy5.5	61D3	Life Technologies
CD16	BUV395	3G8	BD Biosciences
CD19	Percp-Cy5.5	HIB19	BioLegend
CD56	BV421	NCAM16.2	BD Biosciences
CD107a/LAMP1	AF488	H4A3	BioLegend
CD123	Percp-Cy5.5	6H6	BioLegend

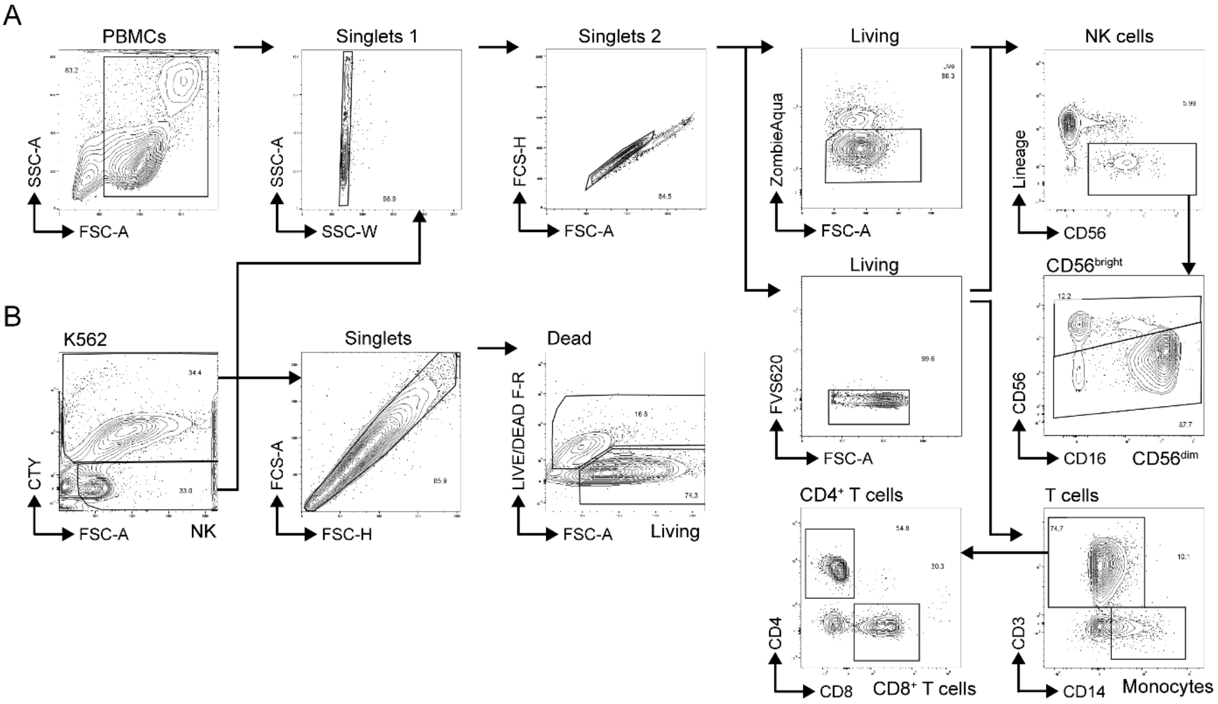

Supplementary Figure 1. Gating strategy of NK cells, monocytes, T cells and K562 target cells. (**A**) Flow cytometric identification of total natural killer (NK) cells as lineage$^-$ (CD3$^-$ CD14$^-$ CD19$^-$ CD123$^-$) CD56$^+$ living singlet PBMCs, with further gating on CD56^{bright} and CD56^{dim} NK cell subsets. T cells were identified as CD3$^+$ living singlet PBMCs with further gating on CD4$^+$ and CD8$^+$ T cell subsets. Monocytes were identified as CD14$^+$ living singlet PBMCs. (**B**) Flow cytometric identification of NK effector cells and K562 tumor cells and their survival in the in vitro NK cell cytotoxicity assay. SSC-A, side scatter area; FSC-A, forward scatter area; SSC-W, side scatter width; FSC-H, forward scatter height; FVS620, Fixable Viability Stain 620; CTY, CellTrace Yellow; LIVE/DEAD F-R, LIVE/DEAD Fixable Far-Red Dead Cell Stain.

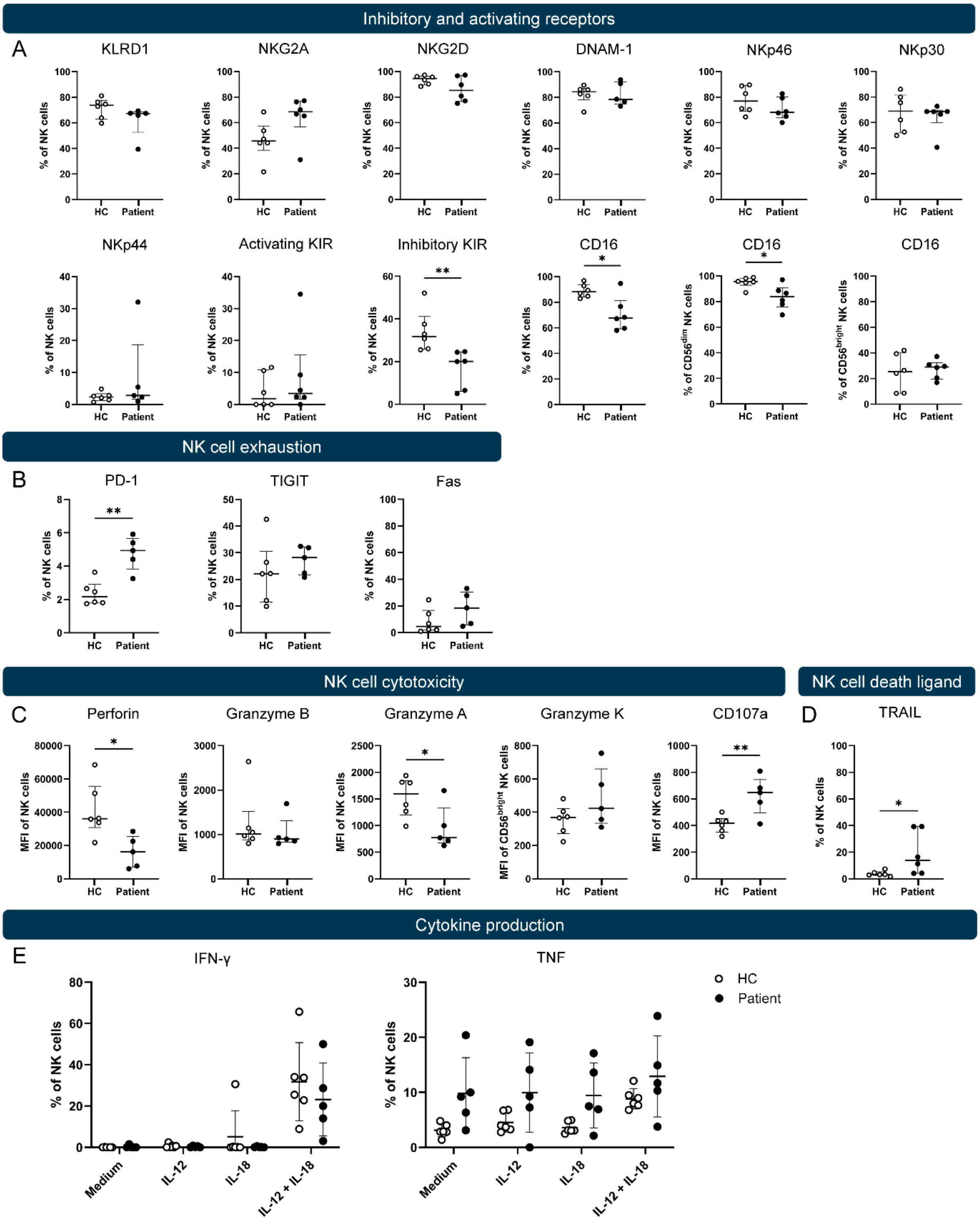

Supplementary Figure 2. Immunophenotyping of NK cells in DADA2. (**A-D**) Scatter plots showing the percentage or median fluorescence intensity (MFI) of inhibitory receptors (KLRD1, NKG2A and inhibitory KIR), activating receptors (NKG2D, DNAM-1, NKp46, NKp30, NKp44, activating KIR and CD16), exhaustion markers and cytotoxic mediators as measured by flow cytometry with each dot representing an individual and horizontal bars showing the median with interquartile range.

*, *P* < 0.05; **, *P* < 0.01 as determined by two-tailed unpaired t-test or two-tailed Mann-Whitney U test (exact test used provided in 'Statistics' section of the methods). (**E**) Scatter plots showing intracellular interferon (IFN)-γ and tumor necrosis factor (TNF) production by NK cells after 14 hour in vitro stimulation and incubation with protein transport inhibitors. Each dot represents an individual and horizontal bars show the median with interquartile range. No statistically significant contrasts were found by a two-way ANOVA with Šídák's multiple comparisons test. Complete gating strategy is shown in Supplementary Figure 1A.

METHODS

Sex as a biological variable

Our study included both male (n = 5) and female (n = 6) patients and sex was not explicitly considered as a biological variable as DADA2 affects both sexes more or less equally. Sex-matched healthy controls (HCs) were included.

Study population

DADA2 patients were diagnosed at the University Hospitals Leuven Primary Immunodeficiency Clinic (Leuven, Belgium) based on clinical phenotype followed by a combination of serum ADA2 enzyme activity and genotyping of the *ADA2* gene according to the DADA2 management guidelines (1). We included a total of 11 DADA2 patients as well as age- and sex-matched HCs. *ADA2* genotype, predominant clinical phenotype and treatment at time of sampling of included patients can be found in Supplementary Table 1. We resampled two patients (P2 and P5) after hematopoietic stem cell transplant (HSCT) for the NK cell degranulation assay. These patients portrayed 90% and 70% of *ADA2* chimerism respectively at time of resampling.

Peripheral blood collection and processing

Peripheral blood was collected into round-bottom lithium heparin tubes (BD Vacutainer). PBMCs were isolated from peripheral blood samples using SepMate density gradient separation (Stemcell Technologies) according to the manufacturer's protocol and resuspended in heat-inactivated fetal bovine serum (FBS, Merck) + 10% dimethyl sulfoxide (DMSO, Merck) for cryopreservation in liquid nitrogen until further processing. For downstream analyses, we resuscitated the cryopreserved PBMCs in warmed RPMI 1640 medium (Gibco) containing 10% FBS. PBMCs were centrifuged for 10 min at 300 g at 6°C and resuspended in PBS for cell count and viability check.

Intracellular detection of IFN-γ and TNF

PBMCs ($0.5x10^6$) were stimulated with recombinant human IL-12 (2 ng/ml, PeproTech) and/or IL-18 (100 ng/ml, MBL) in RPMI 1640 medium (Gibco) containing 10% FBS for 14h at 37°C and 5% CO_2 to induce cytokine production. To accumulate cytokines for detection by flow cytometry, cells were

subsequently incubated with GolgiPlug (1:1000) and GolgiStop (1:1500) (both BD Biosciences) for 4h at 37°C and 5% CO_2.

NK cell degranulation assay (fresh blood)

Freshly isolated PBMCs were seeded (2×10^6/ml) in Iscove's Modified Dulbecco's Medium (ThermoFisher Scientific) supplemented with 10% fetal calf serum and either incubated 1:1 with human erythroleukemia K562 cells (2×10^6/ml) or left in only medium. All conditions were supplemented with anti-CD107a-PE (#555801, BD Biosciences). Twenty hours after in vitro culture (37°C, 5% CO_2), PBMCs were surface-stained with Viability Dye Zombie Violet (Biolegend), anti-CD3 APC-H7 (#560176, BD Biosciences), anti-CD8 V500 (#560774, BD Biosciences) and anti-CD56 APC (#341027, BD Biosciences). Samples were acquired on a FACSymphony A1 Cell Analyzer (BD Biosciences) and analyzed with FlowJo cell analysis software (LLC, V10).

NK cell cytotoxicity assay

PBMCs were resuscitated as described above and allowed to rest for 24h (37°C, 5% CO_2). Next, NK cells were isolated from PBMCs using negative immunomagnetic enrichment (EasySep Human NK Cell Isolation Kit (Stemcell Technologies) according to the manufacturer’s protocol. Isolated NK cells were incubated in RPMI 1640 medium (Gibco) containing 10% FBS and IL-15 (25 ng/ml, PeproTech) overnight at 37°C and 5% CO_2. Human erythroleukemia K562 cells were stained with CellTrace Yellow (ThermoFisher Scientific) according to the manufacturer’s protocol, except for the concentration which we lowered to 31.25 nM. Next, NK cells were incubated with stained K562 cells at a 4:1 (effector:target) ratio (except for 1 patient and 1 healthy control sample where a 2.4:1 ratio was used due to sample limitations) for 4h (37°C, 5% CO_2) in the presence of anti-CD107a-AF488 (BioLegend). One sample contained K562 cells only (0:1 effector:target ratio) to correct for background apoptisis. NK cell – K562 co-cultures were then stained and acquired as described below.

Flow cytometry

Cells were incubated with human FcR-block (Miltenyi Biotec) and Zombie Aqua 516 (1:1000, Biolegend), Fixable Viability Stain 620 (1:8000, BD biosciences) or LIVE/DEAD Fixable Far-Red Dead

Cell Stain (1:32000, Invitrogen) for 15 minutes at RT. Subsequently, cells were incubated with fluorochrome-labeled monoclonal antibodies for 30 minutes at 4° C in the dark (list of human antibodies in Supplementary Table 2). Staining for intracellular proteins was performed with the Cytofix/Cytoperm kit (BD biosciences) or Foxp3/Transcription Factor Staining Buffer Set (Invitrogen), according to the manufacturer's protocol. Flow cytometric analysis was performed on a BD LSR Fortessa X20 with DIVA software. Results were analyzed with FlowJo (LLC, V10). NK cells were gated as lineage$^-$ (CD3$^-$CD14$^-$CD19$^-$CD123$^-$) CD56$^+$ living singlet PBMCs, with further gating on CD56^{bright} and CD56^{dim} NK cell subsets. T cells were identified as CD3$^+$ living singlet PBMCs with further gating on CD4$^+$ and CD8$^+$ T cell subsets. Monocytes were identified as CD14$^+$ living singlet PBMCs (Supplementary Figure 1A). In the NK cell cytotoxicity assay, K562 target cells and NK effector cells were initially discriminated based on the CellTrace Yellow stain and further gating was performed as shown in Supplementary Figure 1B. Further analysis of the NK cell population in all analyses was only performed if the population contained at least 200 cells.

Statistics

GraphPad Prism (v10.3.1) was used for statistical analyses and graphing. Given the small sample size of our cohort and the poor performance of non-parametric tests with such sample sizes, we decided to apply a parametric two-tailed unpaired t-test, provided the data followed a normal distribution according to the Shapiro-Wilk test (perforin; granzyme B on dim; granzyme A on NK and dim; granzyme K on bright; DNAM-1; %bright; %dim; TRAIL on NK and bright; NKG2D; CD16; NKp46; inhibitory KIR; CD57; PD-1; TIGIT; FAS; ULBP on monocytes, CD4+ and T cells). In case normality could not be assumed, we performed a non-parametric two-tailed Mann-Whitney U test (CCR7; HLA-DR; granzyme B on NK and bright; granzyme A on bright; granzyme K on NK and dim; KLRD1; CD49a; CD69; NKp44; CD107a; %NK; NKG2A; TRAIL on dim; NKp30; activating KIR; ULBP on CD8+). For the fresh blood NK cell degranulation graph (Figure 1G), we used a one-way ANOVA with Šídák's multiple comparisons test and for the IFN-γ and TNF-α graphs (Supplementary Figure 2E), we used a two-way ANOVA with Šídák's multiple comparisons test. Size of two-tailed *P* values in the graphs is indicated

as *, $P < 0.05$; **, $P < 0.01$; ***, $P < 0.001$; ****, $P < 0.0001$. Causion is always warranted when interpreting *P* values obtained from small sample sizes.

Study approval

All donors provided written informed consent for peripheral blood collection and data analysis. This study was approved by the Ethics Committee of the University Hospitals Leuven (S63077, S63807) and performed in accordance with the declaration of Helsinki**.**

Data availability

Values for all data points in graphs are reported in the Supporting Data Values file.

Acknowledgements

The authors thank Drs Rik Schrijvers, Lien De Somer and Lieve Sevenants for recruitment of patients and healthy controls. JB holds a PhD Fellowship of the Research Foundation-Flanders (FWO-Vlaanderen, 11A0523N/11A0525N). IM is a Senior Clinical Investigator at the Research Foundation – Flanders (FWO-Vlaanderen) and is supported by the FWO Grant G0B5120N and by the Jeffrey Modell Foundation. This project has received funding from the European Research Council (ERC) under the European Union’s Horizon 2020 research and innovation programme (grant agreement No. 948959). This work is supported by ERN-RITA. Finally, we would like to thank all patients and their families for their contribution to this study.

REFERENCES

1. Lee PY, et al. Evaluation and Management of Deficiency of Adenosine Deaminase 2. *JAMA Netw Open.* 2023;6(5):e2315894.
